# Clinicopathologic Features and Molecular Characteristics of Glucose Metabolism Contributing to ¹⁸F-fluorodeoxyglucose Uptake in Gastrointestinal Stromal Tumors

**DOI:** 10.1371/journal.pone.0141413

**Published:** 2015-10-28

**Authors:** Min-Hee Cho, Cheol Keun Park, Minhee Park, Won Kyu Kim, Arthur Cho, Hoguen Kim

**Affiliations:** 1 Department of Pathology, Yonsei University College of Medicine, Seoul, Korea; 2 Brain Korea 21 PLUS Project for Medical Science, Yonsei University College of Medicine, Seoul, Korea; 3 Department of Nuclear Medicine, Severance Hospital, Yonsei University College of Medicine, Seoul, Korea; Instituto Nacional de Cardiologia, MEXICO

## Abstract

Fluorine-18 fluorodeoxyglucose (^18^F-FDG) positron emission tomography–computed tomography (PET/CT) is useful in the preoperative diagnosis of gastrointestinal stromal tumors (GISTs); however, the molecular characteristics of glucose metabolism of GIST are unknown. We evaluated ^18^F-FDG uptake on preoperative PET/CT of 40 patients and analyzed the expression of glycolytic enzymes in resected GIST tissues by qRT-PCR, western blotting, and immunohistochemistry. Results of receiver operating characteristic curve analysis showed that the maximum standardized uptake value (SUVmax) cut-off value of 4.99 had a sensitivity of 89.5%, specificity was 76.2%, and accuracy of 82.5% for identifying tumors with a high risk of malignancy. We found that ^18^F-FDG uptake correlated positively with tumor size, risk grade, and expression levels of glucose transporter 1 (GLUT1), hexokinase 1 (HK1), and lactate dehydrogenase A (LDHA). Elevated HK and LDH activity was found in high-risk tumors. Among the isoforms of GLUT and HK, GLUT1 and HK1 expression increased with higher tumor risk grade. In addition, overexpression of glycolytic enzymes M2 isoform of pyruvate kinase (PKM2) and LDHA was observed in GISTs, especially in high-risk tumors. These results suggest that upregulation of GLUT1, HK1, PKM2, and LDHA may play an important role in GIST tumorigenesis and may be useful in the preoperative prediction of malignant potential.

## Introduction

Gastrointestinal stromal tumors (GISTs), the most common non-epithelial neoplasms of the gastrointestinal (GI) tract, are defined as “*KIT* or *PDGFRA* mutation-driven mesenchymal tumors that can occur anywhere in the GI tract” [1]. Preoperative diagnosis of GISTs and assessment of their malignant potential are difficult because most GISTs are located in the submucosa. Tumor grading is therefore based primarily on mitotic index and tumor diameter.

The clinical usefulness of ^18^F-fluorodeoxyglucose (FDG) positron emission tomography–computed tomography (PET/CT) has been demonstrated in tumor staging, treatment response assessment, and prognosis prediction in various tumors [2–6]. This technique is also used in tumor staging and evaluation of targeted therapy response in GISTs [7–12]. Many tumors depend on aerobic glycolysis for rapid growth beyond that supported by the vasculature. ^18^F-FDG is an analogue of glucose that allows noninvasive evaluation of the tumor’s glucose metabolism, which can predict treatment response and patient prognosis. ^18^F-FDG enters the cell through glucose transporters (GLUT), is phosphorylated to ^18^F-FDG-6-PO_4_ by hexokinase (HK), and is then “trapped” in the cell as it is not further metabolized, allowing PET/CT acquisition 1 hour after ^18^F-FDG injection. Upregulation of GLUT and HK expression is associated with increased glucose metabolism and ^18^F-FDG uptake in tumors. For example, previous studies have shown that increased GLUT1 expression correlates with higher ^18^F-FDG uptake in lung and breast carcinomas [13–15].

Although ^18^F-FDG PET/CT is useful for the preoperative diagnosis of GISTs, the detailed molecular mechanisms underlying glucose metabolism in these tumors and specific characteristics associated with tumor risk grade are not well understood. Tumor cells consume large amounts of glucose and produce large amounts of lactate compared to normal cells, even in the presence of oxygen. This metabolic switch from oxidative phosphorylation to increased glycolysis (i.e., the Warburg effect) is a common characteristic of malignant tumors [16, 17] and regulated by transcription factors, such as hypoxia inducible factor-1α (HIF-1α), v-myc avian myelocytomatosis viral oncogene homolog (c-Myc), and tumor suppressor p53 (p53) [18–20]. In gastric cancer, ^18^F-FDG accumulation represents tissue hypoxia, rather than GLUT expression [21]. However, analysis of the enzymes involved in glycolysis has not been performed in GISTs, and it is unclear whether the Warburg effect occurs in GISTs. Qualitative and quantitative analysis of glycolytic enzyme expression and their relationship with GIST tumor risk grade may clarify whether the Warburg effect occurs in GISTs. In this study, we aimed to identify 1) the relationship between maximum standardized uptake value (SUVmax) on preoperative ^18^F-FDG PET/CT with GLUT and HK expression in GISTs, 2) the specific isoforms of GLUT and HK that are upregulated according to GIST tumor risk grade, and 3) alterations in the expression of various glycolytic enzymes according to tumor risk grade. By performing this study, we expect to identify the molecular biomarkers predictive of malignant GISTs that can be used in preoperative biopsy or cytology specimens and the molecular mechanisms of GIST detection by ^18^F-FDG PET/CT.

## Materials and Methods

### Patient Selection

Our patient selection criteria specified the inclusion of patients diagnosed with GIST who underwent surgery after preoperative ^18^F-FDG PET/CT, and a total of 40 GIST patients were included in our study. The cases were identified prospectively and consecutively between 2003 and 2013 at Severance Hospital, Yonsei University College of Medicine and from the Liver Cancer Specimen Bank, National Research Resource Bank Program of the Korea Science and Engineering Foundation of the Ministry of Science and Technology. Written informed consent for use of GIST tissues was obtained from all patients, and use of these tissues for research purposes was approved by the Institutional Review Board of Yonsei University of College of Medicine (IRB approval No.4-2015-0227). Risk of malignancy was categorized according to the system described by Fletcher et al [22].

### Mutational analysis

DNA extraction was performed by using the QIAamp DNA FFPE Tissue Kit (Qiagen, GmbH, Hilden, Germany) according to the manufacturer’s instructions. Primer sequences used to amplify v-kit Hardy-Zuckerman 4 feline sarcoma viral oncogene homolog (*KIT*) exons are shown in S1 Table. PCR was carried out using a Veriti thermal cycler (Life Technologies, USA) with the following amplification conditions: 35 cycles of denaturation at 94°C for 30 s, annealing at 60°C for 30 s, and extension at 72°C for 1 min. The amplified products were purified using Agencourt AMPure XP (Beckman Coulter Genomics, Danvers, MA), and direct sequencing was performed using the BigDye Terminator Ready Reaction Cycle Sequencing kit and an ABI Prism 3130 genetic analyzer (Life Technologies).

### PET/CT protocol and quantification

All patients underwent routine ^18^F-FDG PET/CT scans with either the DSTe PET/CT scanner (GE Healthcare, Milwaukee, WI) or the Biograph TruePoint 40 PET/CT scanner (Siemens Medical Systems, CTI, Knoxv/ille, TN). Before ^18^F-FDG injections, all patients fasted for at least 6 hours, and peripheral blood glucose levels were confirmed to be ≥140 mg/dL. The ^18^F-FDG dose of approximately 5.5 MBq/kg body weight was administered intravenously 1 hour before image acquisition. After the initial low-dose CT (DSTe: 30mA, 130 kVp, Biograph TruePoint: 36 mA, 120 kVp), standard PET imaging was performed from neck to the proximal thighs (acquisition time, 3 min/bed) in three-dimensional mode. Images were then reconstructed using ordered-subset expectation maximization (2 iterations, 20 subsets). Written informed consent was obtained from all patients undergoing ^18^F-FDG PET/CT scans.

Images were reviewed by an experienced nuclear medicine specialist on a GE AW 4.0 workstation. On PET scans, a volume of interest (VOI) was drawn on the primary lesion; SUVmax of the GIST was recorded.

### Immunohistochemistry

Formalin-fixed and paraffin-embedded GIST tissue specimens were cut into 4-μm thick sections, and immunohistochemistry (IHC) analysis was performed using the Ventana Discovery XT autoimmunostainer (Ventana, Tucson, AZ) with antibodies against GLUT1 (1:100; Millipore, Temecula, CA), GLUT3 (1:100; Proteintech, Chicago, IL), GLUT4 (1:200; Abcam, Cambridge, MA), HK1 (1:800; Cell Signaling Technology, Beverly, MA), HK2 (1:500; Cell Signaling Technology), PKM2 (1:500; Cell Signaling Technology), LDHA (1:100; Cell Signaling Technology), HIF-1α (1:100; Novus Biologicals, Littleton, CO), p53 (1:100; Dako, Glostrup, Denmark), and c-Myc (1:100; Santa Cruz Biotechnology, Santa Cruz, CA). IHC results were scored based on staining intensity as follows: 0, no staining; 1, weak staining (faint protein expression in tumor cells or definite expression in <30% of tumor cells); or 2, strong staining (definite protein expression in >30% of tumor cells).

### Western blotting

Whole lysates from GIST tissues were prepared using passive lysis buffer (Promega, Madison, WI) with a protease inhibitor cocktail (Roche, Mannheim, Germany). Total protein lysates (5 μg) were loaded into each lane, size-fractionated by SDS-PAGE and transferred to a nitrocellulose membrane that was blocked with Tris-buffered saline-Tween 20 containing 5% skim milk. Primary antibodies against GLUT1 (1:500; Millipore), HK1 (1:1000; Cell Signaling Technology), PKM2 (1:1000; Cell Signaling Technology), LDHA (1:1000; Cell Signaling Technology), β-actin (1:2000; Cell Signaling Technology), and glyceraldehyde 3-phosphate dehydrogenase (GAPDH, 1:100,000; Trevigen, Gaitherburg, MD) were incubated with the membrane for overnight at 4°C. After washing, membranes were incubated with goat anti-rabbit or mouse IgG-HRP conjugated secondary antibody (Santa Cruz Biotechnology, Santa Cruz, CA), washed, and then developed using western blotting luminol reagent (Santa Cruz Biotechnology). Protein band intensity was analyzed by using a LAS-4000 Mini camera (Fujifilm, Tokyo, Japan).

### Quantitative reverse transcription-polymerase chain reaction

Primer sequences are listed in S2 Table. Quantitative reverse transcription-polymerase chain reaction (qRT-PCR) analysis was carried out in a final reaction volume of 20 μl with Premix Ex Taq II (Takara Bio, Otsu, Japan) according to the manufacturer’s protocol. All reactions were run in triplicate on the StepOnePlus Real-Time PCR System (Applied Biosystems, Foster City, CA).

### Hexokinase and lactate dehydrogenase activity assay

Hexokinase (HK) and lactate dehydrogenase (LDH) activities in GIST tissues were determined using a colorimetric (450 nm) kit (Sigma-Aldrich, St Louis, MO) according to the manufacturer’s recommended protocol. Each sample was diluted as necessary to fall in the linear range of the standard curve and assayed in duplicate. Briefly, 10 μl samples were mixed with assay buffer in HK activity assay and 5 μl samples were mixed with assay buffer in LDH activity assay. Absorbance was measured at room temperature every 5 minutes (HK activity) and at 37°C every 2–3 minutes (LDH activity). Both enzyme activities were assessed using the following equation: *B* × sample dilution factor/(*T*_*final*_ *—T*_*initial*_) × *V;* where *B* is the amount of NADH generated between *T*_*initial*_ and *T*_*final*_, and (*T*_*final*_ *—T*_*initial*_) indicates the reaction time. *V* is the sample volume (mL) added to the reaction well. All data were expressed as milliunit per mg of total protein. One unit of HK and LDH is the amount of enzyme that generate 1 μmole of NADH per minute.

### Statistical analysis

Relationships between tumor risk grade and other parameters were evaluated using either chi-square test or one-way analysis of variance. Correlation analysis was performed with Pearson’s or Spearman’s correlation test. Student’s *t*-test was used to compare two groups of continuous variables. The area under the receiver operating characteristic (ROC) curve was used to determine the SUV cut-off level able to predict tumor risk grade with the highest sensitivity. Data are expressed as mean ± standard deviation (SD); *P* < .05 was considered significant. Statistical analysis was performed using SPSS for Windows (version 21.0; SPSS Inc., USA).

## Results

### Clinicopathologic characteristics and ^18^F-FDG uptake of 40 GISTs

**Fig 1:**
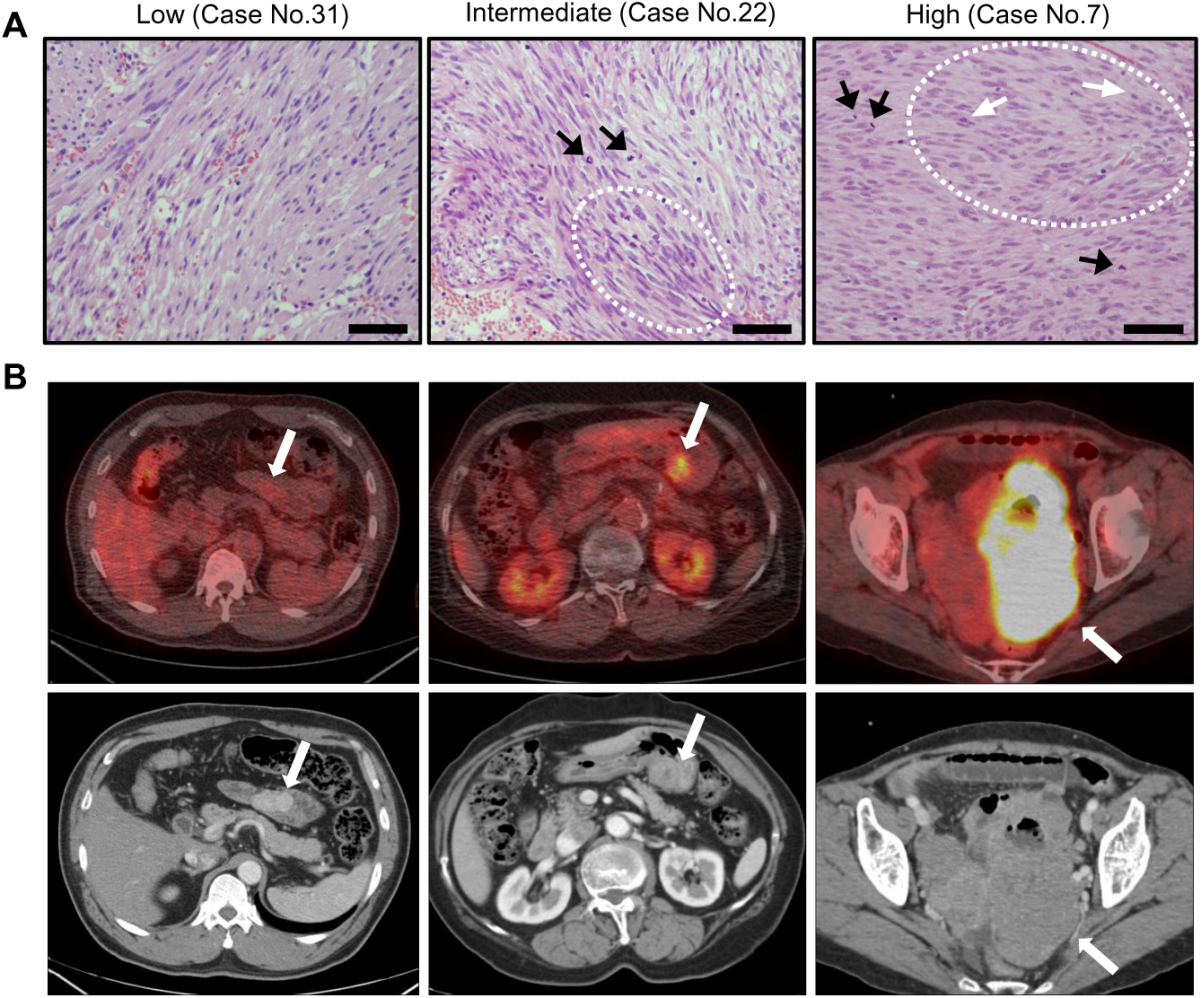
Histologic features of GISTs and Representative cases showing ^18^F-FDG uptake intensity according to tumor risk grade. (A) Low-risk GIST showing proliferation of bland-looking spindle cells with infrequent mitoses (1/50 HPFs) (left panel). Intermediate-risk GIST showing mild nuclear pleomorphism (dashed line) with occasional mitosis (10/50 HPFs; black arrows) (middle panel). High-risk GIST showing proliferation of spindle cells with hyperchromatic nuclei (white arrows), moderate pleomorphism, and frequent mitoses (13/50 HPFs) (right panel). Scale bar, 100μm; ×200. (B) Low-risk GIST: 58-year-old man with mild ^18^F-FDG uptake (SUVmax = 2.2) in the proximal jejunum (left panel). Intermediate-risk GIST: 73-year-old woman with moderate ^18^F-FDG uptake (SUVmax = 4.9) (middle panel). High-risk GIST: 65-year-old woman with intense ^18^F-FDG uptake (SUVmax = 19.2) (right panel).

Of the 40 patients included in the study, 22 were women and 18 were men (S3 Table), and mean patient age was 59 years (range 20–83). Of the 40 GIST lesions, 18 were located in the stomach, 22 were located in the small or large intestine. Five lesions were not well visualized on PET (SUVmax<2.5), 12 showed moderate FDG uptake (2.5<SUVmax<5.0), and 23 showed intense FDG uptake (SUVmax≥5.0). High-risk tumors were more common in men (Table 1). Histologic features of representative cases are shown in Fig 1A.

**Table 1:** Relationships between GIST risk grade and ^18^F-FDG uptake and demographic and clinicopathologic factors.

Variable category	Tumor risk grade			
	Low (n = 13)	Intermediate(n = 8)	High (n = 19)	*P* value
**Age, years**	57.5±13.3	61.5±19.4	58.4±12.5	0.814
**Gender, n**				
**Male**	5	1	12	0.046*
**Female**	8	7	7	
**Tumor size, cm**	3.2±1.0	5.9±2.1	9.6±4.0	< .001*
**Mitotic count, /50 HPF**	2.1±1.3	4.9±3.5	27.5±35.2	0.013*
**SUVmax**	4.3±3.2	5.4±2.7	10.9±5.2	< .001*

Results are expressed as mean±SD unless otherwise indicated.

*P value was calculated either by chi-square test or one-way analysis of variance

FDG uptake did not differ significantly between gastric GISTs and non-gastric GISTs (data not shown); however, SUVmax strongly correlated with tumor size (r = 0.716, *P* < .001). In addition, SUVmax was lower in tumors with low mitotic count (≤10/50 high-power fields [HPF]) than in tumors with high mitotic count (>10/50 HPF) (5.4±3.7 vs. 10.7±5.4, respectively; *P* < .001) (S1 Fig).

### Correlation between ^18^F-FDG uptake and tumor risk grade

Tumor risk grade [22] correlated significantly with ^18^F-FDG uptake (*P* < .001) (Table 1 and Fig 1B). SUVmax was lower for low-risk (4.3±3.2) and intermediate-risk tumors (5.4±2.7) than for high-risk tumors (10.9±5.2; *P* < .001).

To evaluate the usefulness of ^18^F-FDG PET/CT in predicting the malignant potential of GISTs, we compared high-risk tumors with intermediate- and low-risk tumors. ROC curve analysis showed that a SUVmax cut-off of 4.99 was the most sensitive for predicting malignancy, and area under curve was 0.875 (*P* < .001). Using this SUVmax cut-off value to differentiate high-risk tumors from low- and intermediate-risk tumors, sensitivity was 89.5% (16/18), specificity was 76.2% (17/22), and accuracy was 82.5% (32/40).

### Overexpression of GLUT1 and HK1 in GISTs according to tumor risk grade

**Fig 2:**
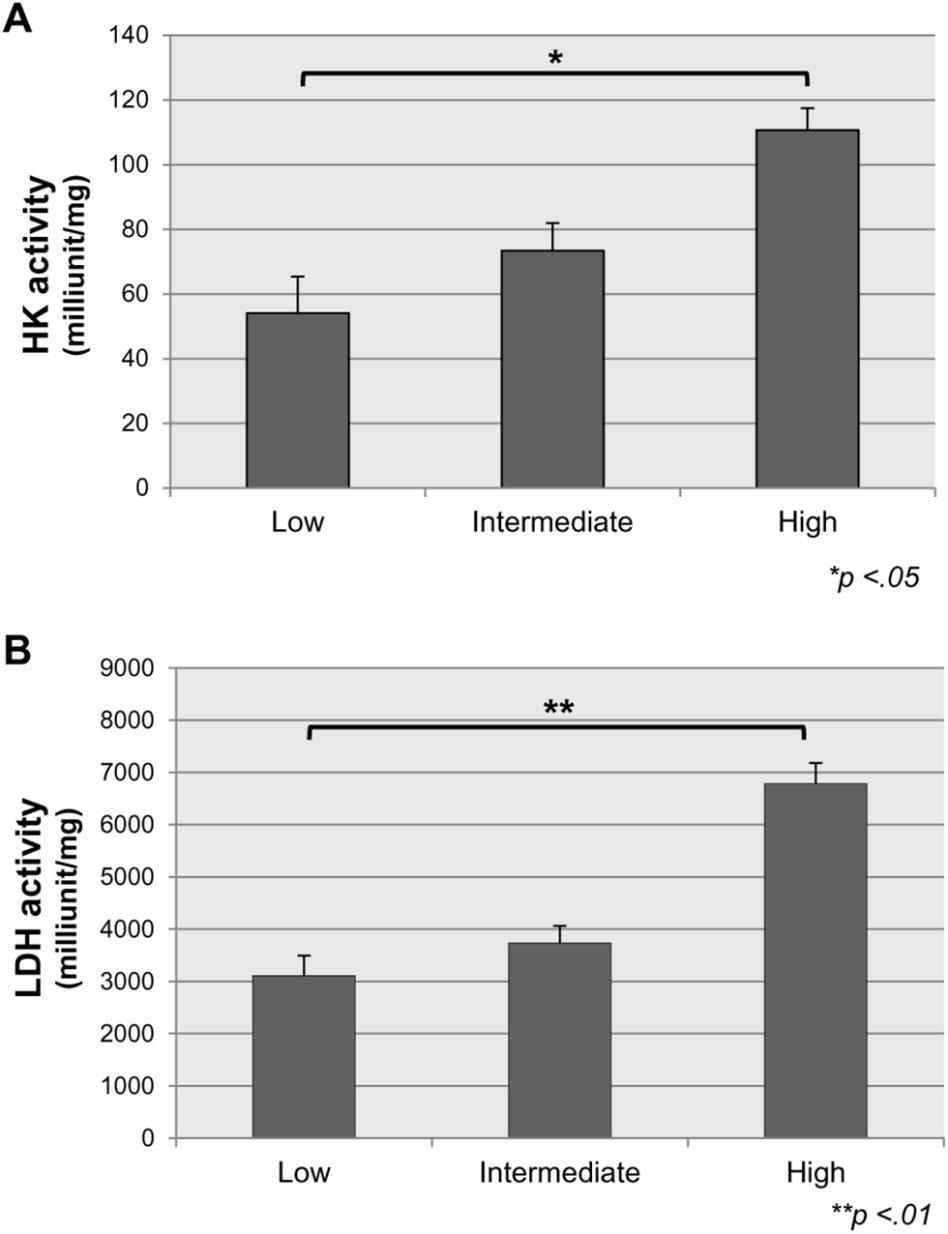
HK and LDH activity in GISTs. (A) (B). Elevated HK and LDH activities are found in high-grade GISTs. Results are expressed as mean±SD of three independent experiments. *P < .05; **P < .01 based on the Student’s *t*-test.

**Fig 3:**
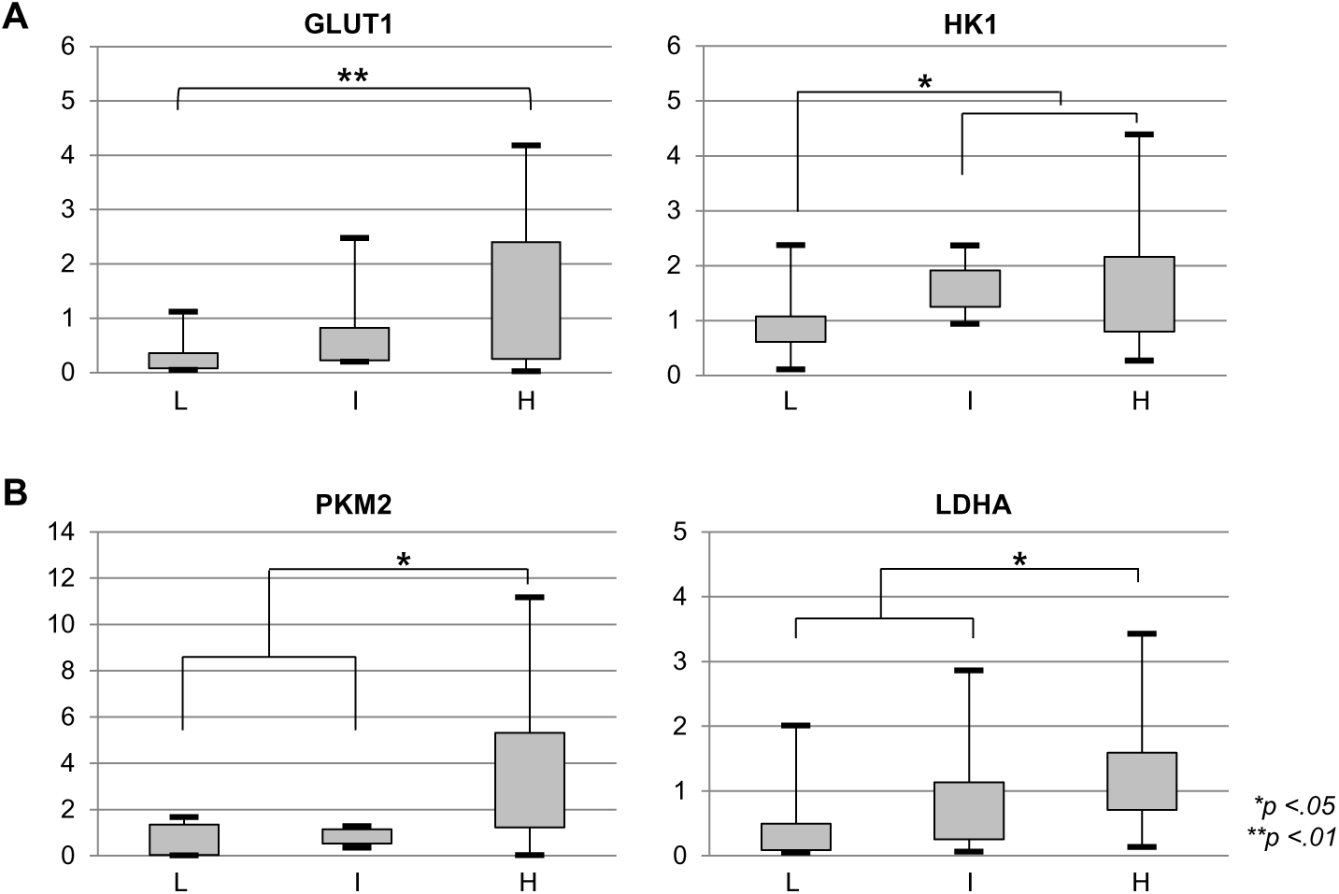
*GLUT1*, *HK1*, *PKM2*, and *LDHA* expression according to GIST risk grade, as assessed by quantitative RT-PCR. (A) (B). *GLUT1*, *HK1*, *PKM2*, and *LDHA* mRNA levels increased with higher tumor risk grade. L = low risk; I = intermediate risk; H = high risk. *P < .05; **P < .01 based on the Student’s *t*-test.

Because isoforms of GLUT and HK are overexpressed in tumors and associated with ^18^F-FDG uptake, we evaluated the expression of four isoforms of GLUT (GLUT 1, 2, 3, and 4) and two isoforms of HK (HK1 and HK2) in the 40 GISTs. First, we analyzed total HK activity. We found that HK activity was significantly upregulated in high-risk GISTs, compared to low-risk and intermediate-risk GISTs (P < .05) (Fig 2A). Results of qRT-PCR analysis showed gradual increases in *GLUT1 and HK1* expression with higher tumor risk grade (Fig 3A). In contrast, expression of *GLUT 2*, *3*, *4*, and *HK2* was not correlated with tumor risk grade at both the mRNA and protein level (S2A and S2B Fig). Expression of *GLUT1* was significantly increased in high-risk GISTs (1.3±1.32) compared to low-risk GISTs (0.27±0.32; *P* < .01). Similarly, *HK1* expression was increased in intermediate-risk (1.65±0.48) and high-risk GISTs (1.58±1.10) compared to low-risk GISTs (0.94±0.68; *P* < .05).

**Fig 4:**
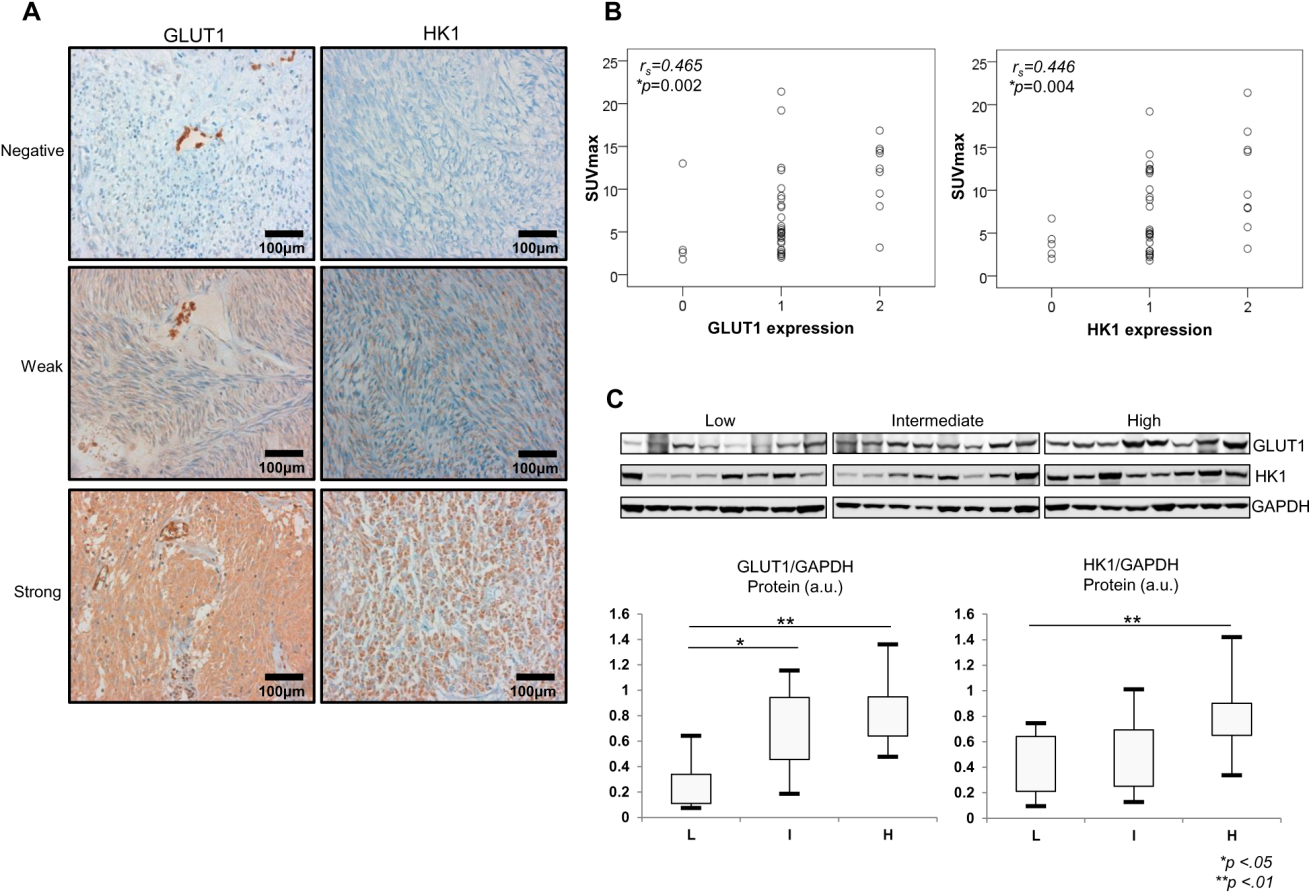
SUVmax correlated with GLUT1 and HK1 expression. (A) Immunohistochemical analysis of GLUT1 and HK1 expression in representative tumor tissues (×200). (B) Correlation between ^18^F-FDG uptake and GLUT1 and HK1 expression (*r*_*s*_ = Spearman’s correlation coefficient). (C) Western blotting and densitometric analysis of GLUT1 and HK1 expression in representative tissues. The densitometric data are normalized to GAPDH and shown as arbitrary units. L = low risk; I = intermediate risk; H = high risk. *P < .05; **P < .01 based on the Student’s *t*-test.

We next analyzed GLUT1 and HK1 protein expression by IHC and western blot analysis. GLUT1 and HK1 protein were detected in most GISTs; however, GLUT1 was not detected in four GISTs, and HK1 was not detected in five GISTs (Table 2). Most of the tumors lacking expression of these two proteins were low-risk GISTs (Table 2 and Fig 4A). Results of IHC staining showed that SUVmax correlated with both GLUT1 expression (*r*_*s*_ = 0.465, *P* = 0.002) and HK1 expression (*r*_*s*_ = 0.446, *P* = 0.004) (Fig 4B). These findings suggest that GLUT1 and HK1 expression are related to PET signals. Results of western blotting analysis showed expression patterns of GLUT1 and HK1 that were similar to qRT-PCR and IHC results (Fig 4C).

**Table 2:** Relationships between GLUT1, HK1, PKM2, and LDHA immunohistochemical expression and GIST tumor risk grade.

Category	Tumor risk grade			
	Low (n = 13)	Intermediate (n = 8)	High (n = 19)	*P* value
**GLUT1, n (%)**				
**Negative**	3 (23.1)	0 (0.0)	1 (5.3)	0.024*
**Weak**	10 (76.9)	7 (87.5)	10 (52.6)	
**Strong**	0 (0.0)	1 (12.5)	8 (42.1)	
**HK1, n (%)**				
**Negative**	4 (30.8)	0 (0.0)	1 (5.3)	0.036*
**Weak**	9 (69.2)	6 (75.0)	11 (57.9)	
**Strong**	0 (0.0)	2 (25.0)	7 (36.8)	
**PKM2, n (%)**				
**Negative**	6 (46.2)	1 (12.5)	1 (5.3)	0.02*
**Weak**	7 (53.8)	6 (75.0)	12 (63.2)	
**Strong**	0 (0.0)	1 (12.5)	6 (31.6)	
**LDHA, n (%)**				
**Negative**	0 (0.0)	0 (0.0)	0 (0.0)	0.016*
**Weak**	10 (76.9)	3 (37.5)	5 (26.3)	
**Strong**	3 (23.1)	5 (62.5)	14 (73.7)	

*P value was calculated either by chi-square test or Fisher’s exact test.

### Enhanced expression of PKM2 and LDHA in high-risk GISTs

In order to elucidate the presence of the Warburg effect in GISTs, we evaluated mRNA and protein expression levels for PKM2 and LDHA. Results of qRT-PCR showed a gradual increase in *PKM2* and *LDHA* expression with higher tumor risk grade (Fig 3B). *PKM2* expression was significantly higher in high-risk GISTs (3.39±3.51) than in low-risk (0.69±0.67) and intermediate-risk GISTs (0.85±0.36; *P* < .05). Similarly, *LDHA* expression was significantly higher in high-risk GISTs (1.37±1.09) than in low-risk (0.49±0.63) and intermediate-risk GISTs (0.89±0.92; *P* < .05).

**Fig 5:**
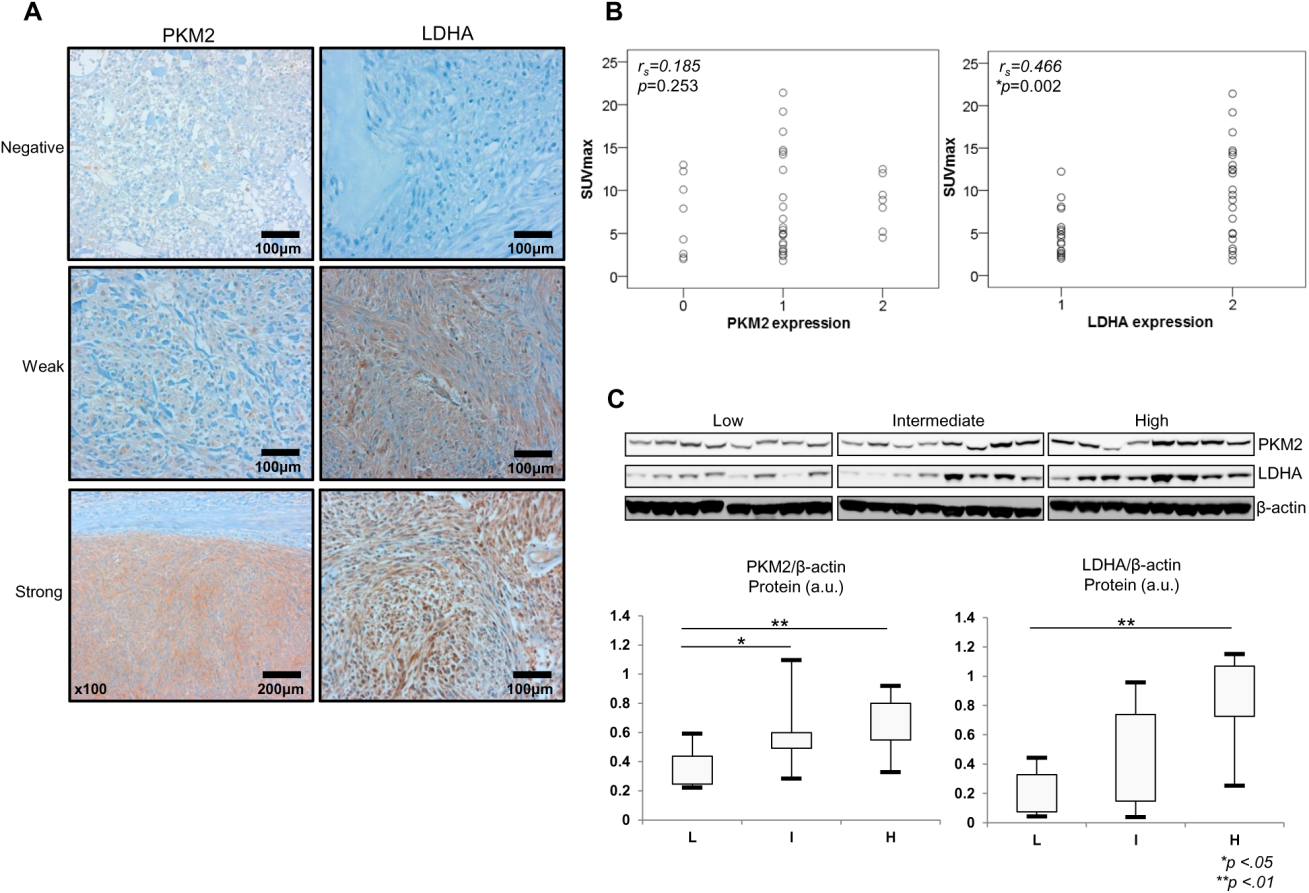
SUVmax correlated with LDHA expression. (A) Immunohistochemical analysis of PKM2 and LDHA expression in representative tumor tissues. (B) Correlation between ^18^F-FDG uptake and PKM2 and LDHA protein expression (*r*_*s*_ = Spearman’s correlation coefficient). (C) Western blotting and densitometric analysis of PKM2 and LDHA expression in representative tissues. The densitometric data are normalized to β-actin and shown as arbitrary units. *P < .05; **P < .01 based on the Student’s *t*-test.

Next, we evaluated protein levels of PKM2 and LDHA in the 40 GISTs by IHC and western blotting analysis. These two glycolytic enzymes were detected in most GISTs; however, PKM2 was not expressed in eight GISTs (Table 2). Most of the tumors lacking protein expression of these enzymes were low-risk GISTs. Protein levels of PKM2 and LDHA were increased in intermediate- and high-risk GISTs (Table 2 and Fig 5A). We also found a positive correlation between SUVmax and LDHA protein expression. SUVmax was significantly higher in tumors with strong LDHA expression than in tumors with weak LDHA expression (*r*_*s*_ = 0.466, *P* = 0.002) (Fig 5B). Results of western blotting analysis showed that PKM2 and LDHA expression patterns were similar to IHC results (Fig 5C). Additionally, we measured LDH activity in our GIST tissues to evaluate metabolism status. LDH activity was significantly upregulated in high-risk GISTs, compared to low-risk and intermediate-risk GISTs (*P* < .01) (Fig 2B). These findings suggest that upregulation of LDHA is involved in the malignant potential and PET signal of GISTs.

We further evaluated the relationship between the glycolysis-modulating transcription factors and glycolytic markers that we had selected (GLUT1, HK1, PKM2, and LDHA). First, we analyzed HIF-1α, c-Myc, and p53 expression using qRT-PCR and IHC. We found that mRNA expression levels of *HIF-1α* were higher in high-risk GISTs (1.38±0.80) than in low-risk (0.66±0.22) and intermediate-risk GISTs (1.02±0.63; *P* < .05). The expression levels of *c-Myc* and *p53*, however, did not differ according to GIST risk grade (S3A Fig). IHC staining revealed positive HIF-1α expression in 24 lesions (24/40; 60.0%); 14 of these 24 lesions (58.3%) were high-risk GISTs. All of the GIST specimens were positive for c-Myc, an oncogenic transcription factor. In contrast, expression of p53, a tumor suppressor, was negative in 38 lesions (95.0%) (S3B Fig). Analyzing the correlations between each of the glycolytic markers and these transcription factors, we found that only *HIF-1α* expression showed moderated correlation with *GLUT1* (*r* = 0.390, *P* < .05), *HK1* (*r* = 0.406, *P* < .05), and *LDHA* (*r* = 0.336, *P* < .05) at the mRNA level (S4 Fig).

## Discussion

In this study, we evaluated clinicopathologic factors contributing to ^18^F-FDG uptake in the GISTs of 40 consecutive patients. Positive correlations were found between ^18^F-FDG uptake and several clinicopathologic features and metabolic factors. SUVmax correlated with tumor size; expression of GLUT1, HK1, and LDHA; and NIH risk group. ^18^F-FDG uptake was also significantly higher in GISTs with mitotic count >10/50 HPF compared to GISTs with lower mitotic count. The optimal SUVmax cut-off value for identifying tumors with a high risk of malignancy (NIH risk classification) was 4.99 (sensitivity, 89.5%; specificity, 76.2%). These findings suggest that PET/CT may be useful for preoperative assessment of malignant potential.

We demonstrated significant overexpression of GLUT1 and HK1 protein in high-risk GISTs by IHC and western blot analysis. We also demonstrated that HK activity in upregulated in high-risk GISTs. GLUT and HK are important for glucose uptake, and the GLUT family of transporters has been implicated in ^18^F-FDG uptake, with GLUT1 and GLUT3 in particular playing important roles in ^18^F-FDG accumulation [13, 23]. The role of HK in ^18^F-FDG uptake has been studied in various tumors [15, 24, 25]. In our study, the isoforms GLUT1 and HK1 were specifically overexpressed in high-risk GISTs, however, the expression levels of GLUT2, 3, 4, and HK2 showed no relationship with GIST risk grade. Overexpression of GLUT1 in the cell membrane had been reported in many tumors, and overexpression of GLUT2 and GLUT3 has been reported in hepatocellular carcinoma and malignant lymphoma, respectively [26, 27]. Although GLUT1, as well as GLUT3 and GLUT4, expression was found in one GIST cell line (GIST-T1) [28], only the expression of GLUT1 was related to GIST risk grade. The paired overexpression of GLUT1 and HK1 in GISTs, and the correlation between increased overexpression of these two proteins and tumor risk grade provide evidence for increased glucose uptake and abnormal glucose metabolism in GISTs, which may be useful in preoperative diagnosis and the development of novel therapeutic targets.

In this study, we demonstrated that the Warburg effect exists in GISTs and observed a correlation between ^18^F-FDG uptake and tumor risk grade. Most GISTs evaluated in this study showed significant overexpression of the glycolytic enzymes PKM2 and LDHA, with the degree of overexpression increasing with higher tumor risk grade. We tried to identify which transcription factors induce glycolytic markers in GISTs and found moderate correlation between HIF-1α expression and GLUT1, HK1, and LDHA expression. This metabolic switch from oxidative phosphorylation to increased glycolysis is one of the principle biochemical characteristics of malignant cells [16]. In addition, PKM2 possesses protein tyrosine kinase activity and plays a role in modulating gene expression, thereby contributing to tumorigenesis [29]. For example, enhanced PKM2 expression correlates with aggressive tumor behavior (in vivo tumor growth, tumor cell proliferation, migration) in colon cancer [30]. LDHA expression is also elevated in many types of cancers and is linked to tumor growth, maintenance, and invasion [31–34]. Therefore, LDHA inhibition may restrict the energy supply in cancer cells, thereby decreasing their tumorigenicity [24, 35]. This enzyme may also be useful as a diagnostic marker or predictive biomarker for many types of cancer, as well as a therapeutic target for new anti-cancer treatments. In addition, the correlation between ^18^F-FDG accumulation and LDHA expression and the possible modulation of ^18^F-FDG uptake through LDHA-AKT-GLUT1 signaling has been reported in lung adenocarcinoma [24]. Based on these reports, our findings showing overexpression of PKM2 and LDHA and the correlation between degree of expression and tumor risk grade in GISTs indicate that overexpression of PKM2 and LDHA may play important roles in GIST tumorigenesis and suggest their usefulness as potential therapeutic targets.

In conclusion, we evaluated the usefulness of preoperative ^18^F-FDG PET/CT for the prediction of malignant potential in GISTs and the relationship between tumor risk grade and the expression of proteins involved in glucose metabolism. Our results showed that ^18^F-FDG uptake correlates with tumor size, tumor risk grade, and expression levels of GLUT1, HK1, and LDHA. The increased expression of GLUT1, HK1, PKM2, and LDHA with higher tumor risk grades indicates important roles for these proteins in GIST tumorigenesis and suggests their usefulness in the preoperative prediction of malignant potential.

## Supporting Information

S1 Fig Relationships between SUVmax and tumor size and mitotic count. *r* = Pearson’s correlation coefficient.
(TIF)

S2 Fig Expression levels of GLUT2, 3, 4, and HK2 in GISTs, as assessed by quantitative RT-PCR (A) and immunohistochemistry (B). (TIF)

S3 Fig Expression levels of HIF-1α, c-Myc, and p53 in GISTs, as assessed by quantitative RT-PCR (A) and immunohistochemistry (B). *P < .05 based on the Student’s *t*-test.
(TIF)

S4 Fig Correlation analysis between *HIF-1α* and *GLUT1*, *HK1*, *LDHA*, and *PKM2* mRNA expression. *r* = Pearson’s correlation coefficient.
(TIF)

S1 Table Primer sequences for PCR amplification of the *KIT* gene. (DOCX)

S2 Table Primer sequences for qRT-PCR. (DOCX)

S3 Table Demographic and clinicopathologic features of 40 patients with GISTs. (DOCX)

## References

[1] Miettinen M, Lasota J. Gastrointestinal stromal tumors: review on morphology, molecular pathology, prognosis, and differential diagnosis. Arch Pathol Lab Med. 2006;130(10):1466–78. 10.1043/1543-2165(2006)130[1466:GSTROM]2.0.CO;2 . 17090188

[2] Miller TR, Pinkus E, Dehdashti F, Grigsby PW. Improved prognostic value of 18F-FDG PET using a simple visual analysis of tumor characteristics in patients with cervical cancer. J Nucl Med. 2003;44(2):192–7. . 12571208

[3] de Geus-Oei LF, Vriens D, van Laarhoven HW, van der Graaf WT, Oyen WJ. Monitoring and predicting response to therapy with 18F-FDG PET in colorectal cancer: a systematic review. J Nucl Med. 2009;50 Suppl 1:43S–54S. 10.2967/jnumed.108.057224 . 19403879

[4] Tagliabue L, Del Sole A. Appropriate use of positron emission tomography with [(18)F]fluorodeoxyglucose for staging of oncology patients. Eur J Intern Med. 2014;25(1):6–11. 10.1016/j.ejim.2013.06.012 . 23910561

[5] Shreve P, Faasse T. Role of positron emission tomography-computed tomography in pulmonary neoplasms. Radiol Clin North Am. 2013;51(5):767–79. 10.1016/j.rcl.2013.05.001 . 24010905

[6] Gallamini A, Borra A. Role of PET in lymphoma. Curr Treat Options Oncol. 2014;15(2):248–61. 10.1007/s11864-014-0278-4 . 24619427

[7] Kamiyama Y, Aihara R, Nakabayashi T, Mochiki E, Asao T, Kuwano H, et al 18F-fluorodeoxyglucose positron emission tomography: useful technique for predicting malignant potential of gastrointestinal stromal tumors. World J Surg. 2005;29(11):1429–35. 10.1007/s00268-005-0045-6 . 16222452

[8] Yoshikawa K, Shimada M, Kurita N, Sato H, Iwata T, Morimoto S, et al Efficacy of PET-CT for predicting the malignant potential of gastrointestinal stromal tumors. Surg Today. 2013;43(10):1162–7. 10.1007/s00595-012-0411-6 . 23143169

[9] Otomi Y, Otsuka H, Morita N, Terazawa K, Furutani K, Harada M, et al Relationship between FDG uptake and the pathological risk category in gastrointestinal stromal tumors. J Med Invest. 2010;57(3–4):270–4. . 20847527 10.2152/jmi.57.270

[10] Park JW, Cho CH, Jeong DS, Chae HD. Role of F-fluoro-2-deoxyglucose Positron Emission Tomography in Gastric GIST: Predicting Malignant Potential Pre-operatively. Journal of gastric cancer. 2011;11(3):173–9. 10.5230/jgc.2011.11.3.173 22076223 PMC3204465

[11] Prior JO, Montemurro M, Orcurto MV, Michielin O, Luthi F, Benhattar J, et al Early prediction of response to sunitinib after imatinib failure by 18F-fluorodeoxyglucose positron emission tomography in patients with gastrointestinal stromal tumor. Journal of clinical oncology: official journal of the American Society of Clinical Oncology. 2009;27(3):439–45. 10.1200/JCO.2008.17.2742 . 19064982

[12] Gayed I, Vu T, Iyer R, Johnson M, Macapinlac H, Swanston N, et al The role of 18F-FDG PET in staging and early prediction of response to therapy of recurrent gastrointestinal stromal tumors. J Nucl Med. 2004;45(1):17–21. . 14734662

[13] de Geus-Oei LF, van Krieken JH, Aliredjo RP, Krabbe PF, Frielink C, Verhagen AF, et al Biological correlates of FDG uptake in non-small cell lung cancer. Lung Cancer. 2007;55(1):79–87. 10.1016/j.lungcan.2006.08.018 . 17046099

[14] Chung JK, Lee YJ, Kim SK, Jeong JM, Lee DS, Lee MC. Comparison of [18F]fluorodeoxyglucose uptake with glucose transporter-1 expression and proliferation rate in human glioma and non-small-cell lung cancer. Nucl Med Commun. 2004;25(1):11–7. . 15061260 10.1097/00006231-200401000-00003

[15] Brown RS, Goodman TM, Zasadny KR, Greenson JK, Wahl RL. Expression of hexokinase II and Glut-1 in untreated human breast cancer. Nucl Med Biol. 2002;29(4):443–53. . 12031879 10.1016/s0969-8051(02)00288-3

[16] Warburg O. On the origin of cancer cells. Science. 1956;123(3191):309–14. . 13298683 10.1126/science.123.3191.309

[17] Kim JW, Dang CV. Cancer's molecular sweet tooth and the Warburg effect. Cancer Res. 2006;66(18):8927–30. 10.1158/0008-5472.CAN-06-1501 . 16982728

[18] Marín-Hernández A, Gallardo-Pérez JC, Ralph SJ, Rodríguez-Enríquez S, Moreno-Sánchez R. HIF-1alpha modulates energy metabolism in cancer cells by inducing over-expression of specific glycolytic isoforms. Mini Rev Med Chem. 2009;9(9):1084–101. . 19689405 10.2174/138955709788922610

[19] Miller DM, Thomas SD, Islam A, Muench D, Sedoris K. c-Myc and cancer metabolism. Clin Cancer Res. 2012;18(20):5546–53. 10.1158/1078-0432.CCR-12-0977 23071356 PMC3505847

[20] Archer MC. Role of sp transcription factors in the regulation of cancer cell metabolism. Genes Cancer. 2011;2(7):712–9. 10.1177/1947601911423029 22207896 PMC3218407

[21] Takebayashi R, Izuishi K, Yamamoto Y, Kameyama R, Mori H, Masaki T, et al [18F]Fluorodeoxyglucose accumulation as a biological marker of hypoxic status but not glucose transport ability in gastric cancer. J Exp Clin Cancer Res. 2013;32:34 10.1186/1756-9966-32-34 23718763 PMC3672048

[22] Fletcher CD, Berman JJ, Corless C, Gorstein F, Lasota J, Longley BJ, et al Diagnosis of gastrointestinal stromal tumors: A consensus approach. Hum Pathol. 2002;33(5):459–65. . 12094370 10.1053/hupa.2002.123545

[23] Tian M, Zhang H, Nakasone Y, Mogi K, Endo K. Expression of Glut-1 and Glut-3 in untreated oral squamous cell carcinoma compared with FDG accumulation in a PET study. Eur J Nucl Med Mol Imaging. 2004;31(1):5–12. 10.1007/s00259-003-1316-9 . 14551748

[24] Zhou X, Chen R, Xie W, Ni Y, Liu J, Huang G. Relationship Between 18F-FDG Accumulation and Lactate Dehydrogenase A Expression in Lung Adenocarcinomas. J Nucl Med. 2014;55(11):1766–71. 10.2967/jnumed.114.145490 . 25342384

[25] Park SG, Lee JH, Lee WA, Han KM. Biologic correlation between glucose transporters, hexokinase-II, Ki-67 and FDG uptake in malignant melanoma. Nucl Med Biol. 2012;39(8):1167–72. 10.1016/j.nucmedbio.2012.07.003 . 22901702

[26] Paudyal B, Paudyal P, Oriuchi N, Tsushima Y, Nakajima T, Endo K. Clinical implication of glucose transport and metabolism evaluated by 18F-FDG PET in hepatocellular carcinoma. Int J Oncol. 2008;33(5):1047–54. . 18949368

[27] Shim HK, Lee WW, Park SY, Kim H, So Y, Kim SE. Expressions of glucose transporter Types 1 and 3 and hexokinase-II in diffuse large B-cell lymphoma and other B-cell non-Hodgkin's lymphomas. Nucl Med Biol. 2009;36(2):191–7. 10.1016/j.nucmedbio.2008.11.009 . 19217531

[28] Tanaka M, Kataoka H, Yano S, Ohi H, Moriwaki K, Akashi H, et al Antitumor effects in gastrointestinal stromal tumors using photodynamic therapy with a novel glucose-conjugated chlorin. Mol Cancer Ther. 2014;13(4):767–75. 10.1158/1535-7163.MCT-13-0393 . 24552777

[29] Wong N, De Melo J, Tang D. PKM2, a Central Point of Regulation in Cancer Metabolism. Int J Cell Biol. 2013;2013:242513 10.1155/2013/242513 23476652 PMC3586519

[30] Zhou CF, Li XB, Sun H, Zhang B, Han YS, Jiang Y, et al Pyruvate kinase type M2 is upregulated in colorectal cancer and promotes proliferation and migration of colon cancer cells. IUBMB Life. 2012;64(9):775–82. 10.1002/iub.1066 . 22807066

[31] Rong Y, Wu W, Ni X, Kuang T, Jin D, Wang D, et al Lactate dehydrogenase A is overexpressed in pancreatic cancer and promotes the growth of pancreatic cancer cells. Tumour Biol. 2013;34(3):1523–30. 10.1007/s13277-013-0679-1 . 23404405

[32] Sheng SL, Liu JJ, Dai YH, Sun XG, Xiong XP, Huang G. Knockdown of lactate dehydrogenase A suppresses tumor growth and metastasis of human hepatocellular carcinoma. FEBS J. 2012;279(20):3898–910. 10.1111/j.1742-4658.2012.08748.x . 22897481

[33] Miao P, Sheng S, Sun X, Liu J, Huang G. Lactate dehydrogenase A in cancer: a promising target for diagnosis and therapy. IUBMB Life. 2013;65(11):904–10. 10.1002/iub.1216 . 24265197

[34] Fantin VR, St-Pierre J, Leder P. Attenuation of LDH-A expression uncovers a link between glycolysis, mitochondrial physiology, and tumor maintenance. Cancer Cell. 2006;9(6):425–34. 10.1016/j.ccr.2006.04.023 . 16766262

[35] Le A, Cooper CR, Gouw AM, Dinavahi R, Maitra A, Deck LM, et al Inhibition of lactate dehydrogenase A induces oxidative stress and inhibits tumor progression. Proc Natl Acad Sci U S A. 2010;107(5):2037–42. 10.1073/pnas.0914433107 20133848 PMC2836706

