# Supplementary figures and images for: Clinicopathologic Features and Molecular Characteristics of Glucose Metabolism Contributing to ¹⁸F-fluorodeoxyglucose Uptake in Gastrointestinal Stromal Tumors
Source: PLoS One. 2015 Oct 28;10(10):e0141413. doi: 10.1371/journal.pone.0141413 (PMC4625049; doi:10.1371/journal.pone.0141413)

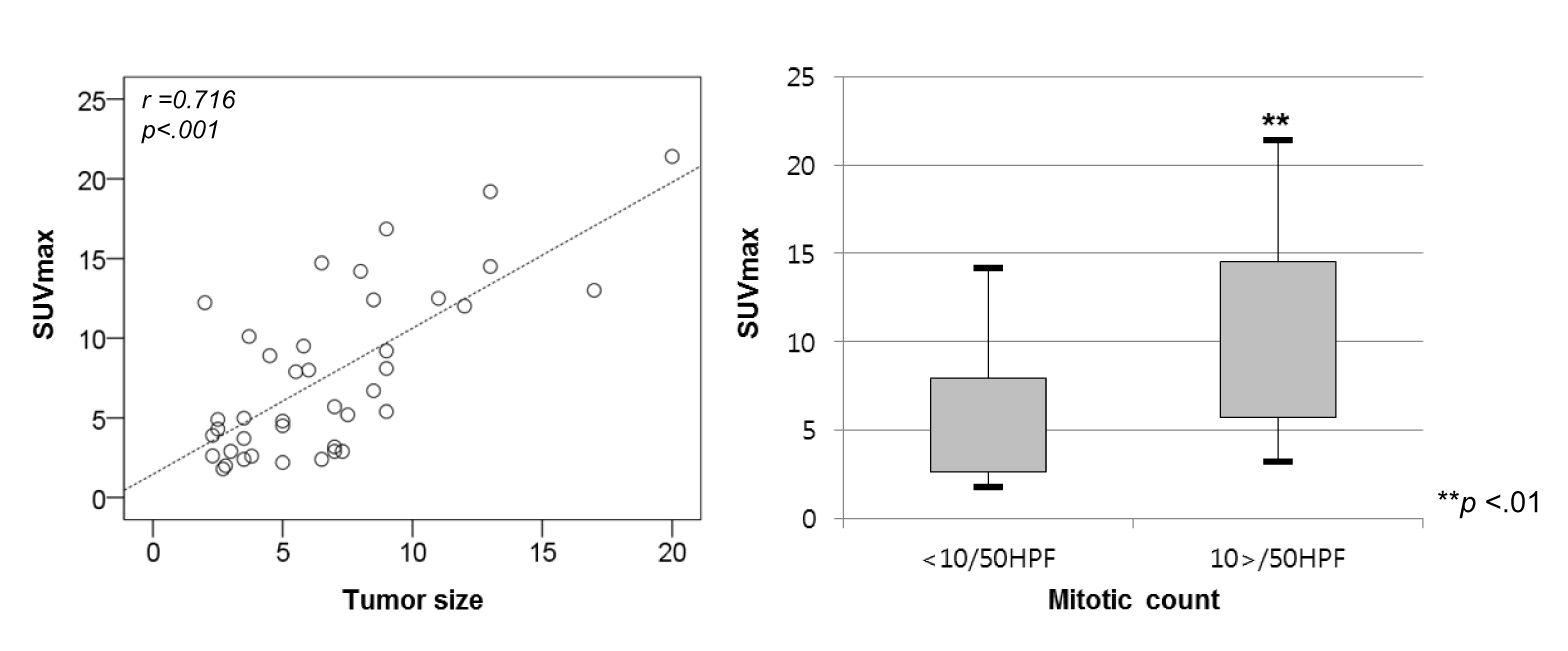

Supplement: S1 Fig — r = Pearson’s correlation coefficient. (TIF) [file pone.0141413.s001.tif]

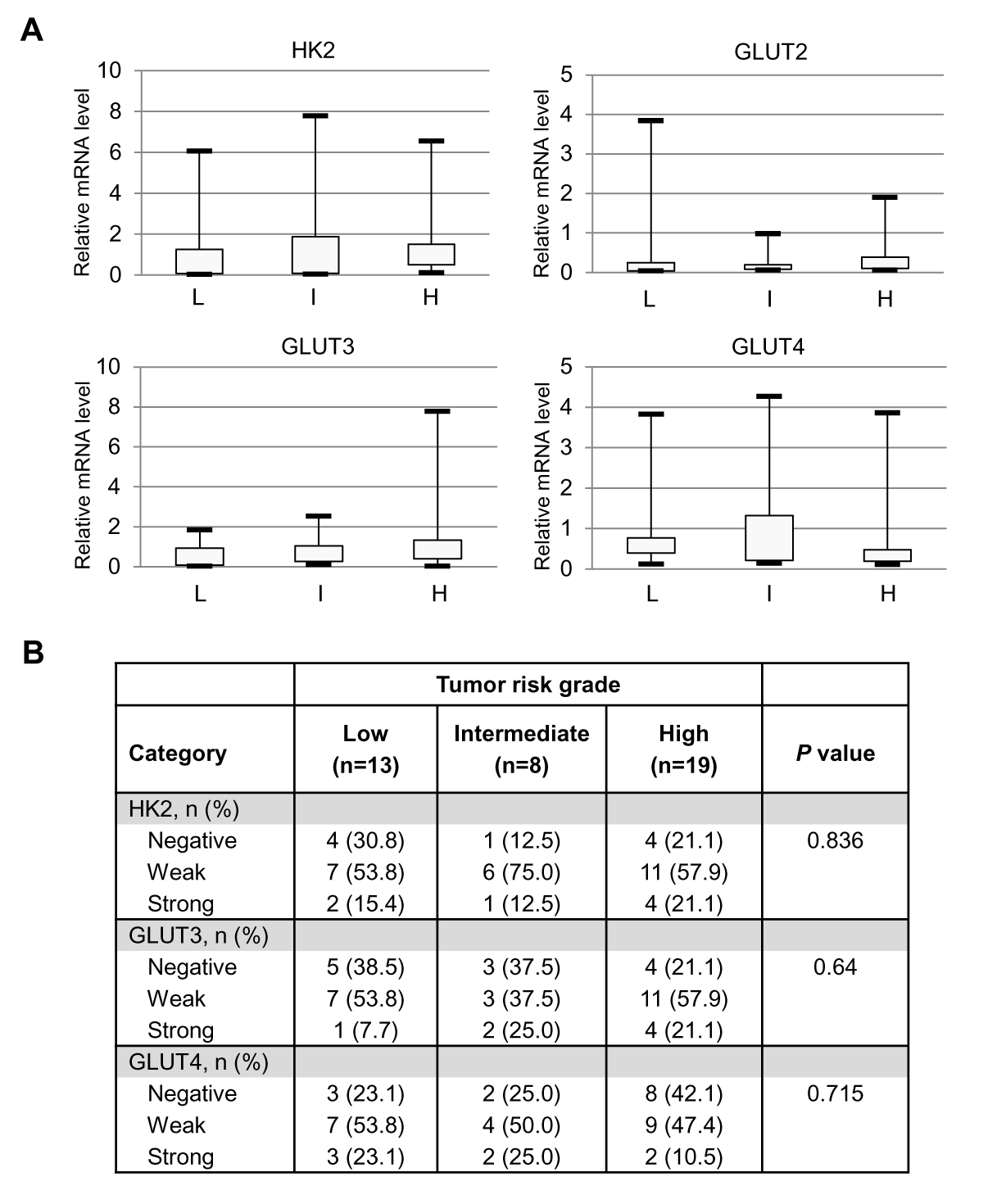

Supplement: S2 Fig — (TIF) [file pone.0141413.s002.tif]

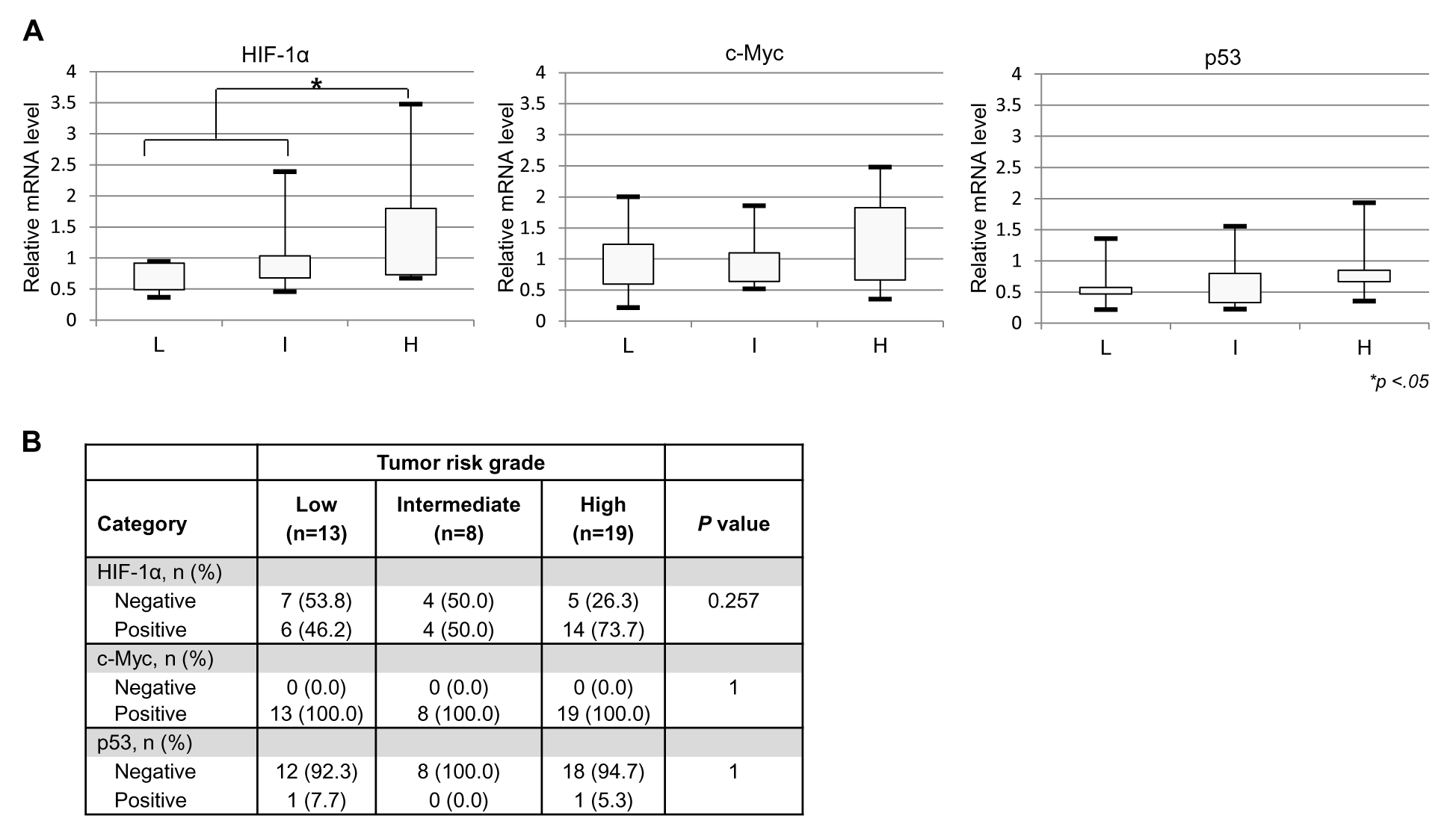

Supplement: S3 Fig — *P < .05 based on the Student’s t-test. (TIF) [file pone.0141413.s003.tif]

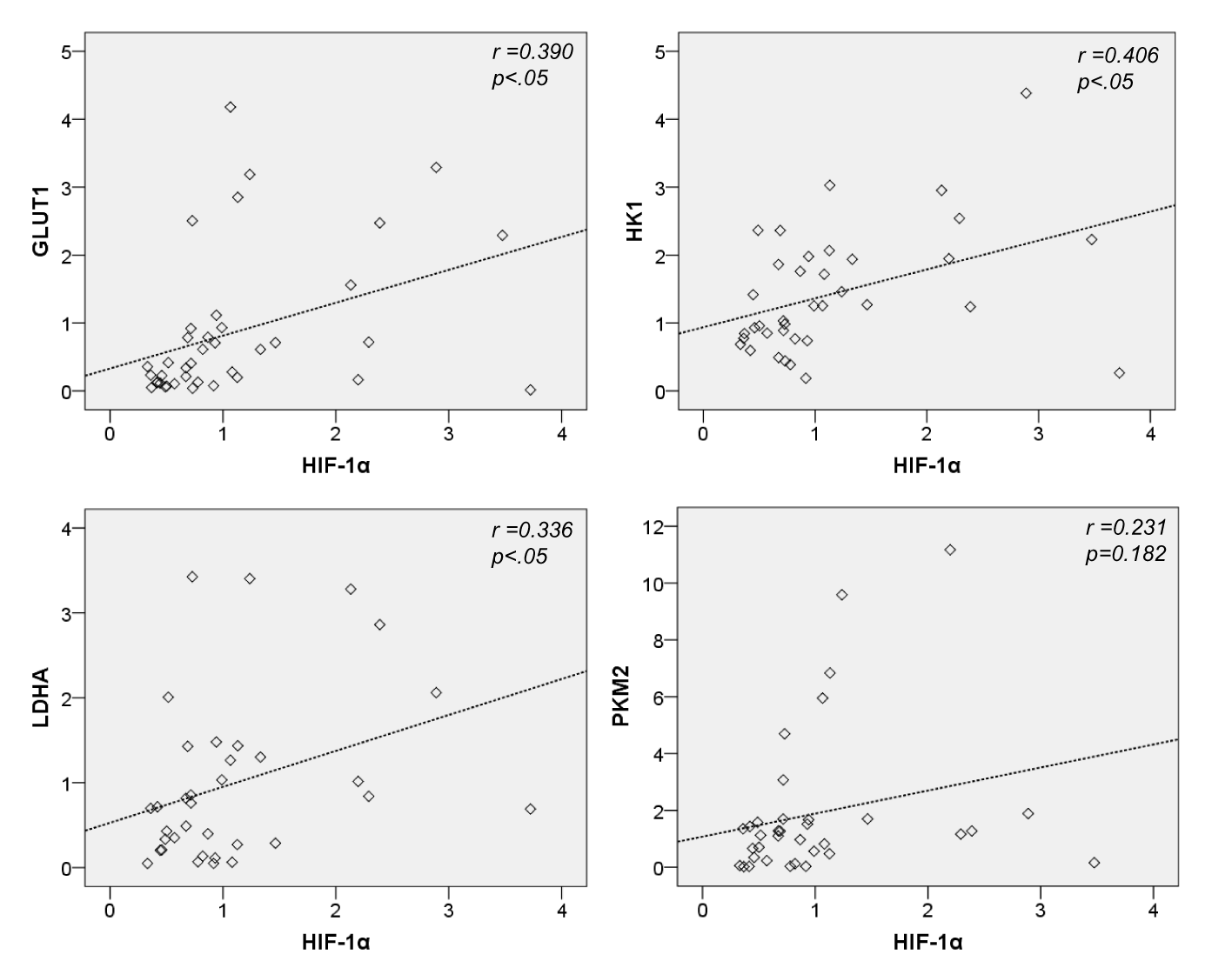

Supplement: S4 Fig — r = Pearson’s correlation coefficient. (TIF) [file pone.0141413.s004.tif]
