# Supplementary material for: Clinicopathologic Features and Molecular Characteristics of Glucose Metabolism Contributing to ¹⁸F-fluorodeoxyglucose Uptake in Gastrointestinal Stromal Tumors
Source: PLoS One. 2015 Oct 28;10(10):e0141413. doi: 10.1371/journal.pone.0141413 (PMC4625049; doi:10.1371/journal.pone.0141413)
Supplement: S1 Table — (DOCX) [file pone.0141413.s005.docx]

**S1 Table.** Primer sequences for PCR amplification of the *KIT* gene.

| Gene | Primer name | Sequence |
| --- | --- | --- |
| *KIT* | Exon9-F | 5’-AGTATGCCACATCCCAAGTG-3’ |
|  | Exon9-R | 5’-TGACTGATATGGTAGACAGAGCC-3’ |
|  | Exon11-F | 5’-GGCATGATGTGCATTATTGTG-3’ |
|  | Exon11-R | 5’-TGGCAAACCTATCAAAAGGG-3’ |
|  | Exon13-F | 5’-ATGCGCTTGACATCAGTTTG-3’ |
|  | Exon13-R | 5’-AAGCAGTTTATAATCTAGCATTGCC-3’ |
|  | Exon17-F | 5’-TGTGAACATCATTCAAGGCG-3’ |
|  | Exon17-R | 5’-AAATGTGTGATATCCCTAGACAGG-3’ |
