# Supplementary material for: Clinicopathologic Features and Molecular Characteristics of Glucose Metabolism Contributing to ¹⁸F-fluorodeoxyglucose Uptake in Gastrointestinal Stromal Tumors
Source: PLoS One. 2015 Oct 28;10(10):e0141413. doi: 10.1371/journal.pone.0141413 (PMC4625049; doi:10.1371/journal.pone.0141413)
Supplement: S2 Table — (DOCX) [file pone.0141413.s006.docx]

**S2 Table.** Primer sequences for qRT-PCR.

| Primer | Sequence | Source |
| --- | --- | --- |
| Beta-actin | F: 5’-GGACCTGACTGACTACCTCAT-3’  R: 5’-CGTAGCACAGCTTCTCCTTAAT-3’ | IDT-Primer Quest |
| GLUT1 | F: 5’-CTCCTGCCCTGTTGTGTATAG-3’  R: 5’-CAGGAGTGAGGTGGTGTATTT-3’ | IDT-Primer Quest |
| GLUT2 | F: 5’-CTAAAGGGCAGGTGGTTCTAAT-3’  R: 5’-TTGCATCCTCAGGTTTCTAGTT-3’ | IDT-Primer Quest |
| GLUT3 | F: 5’-GCTGGGCATCGTTGTTGGA-3’  R: 5’-GCACTTTGTAGGATAGCAGGAAG-3’ | Primer Bank |
| GLUT4 | F: 5’-GGCTTCTTCATCTTCACCTTCT-3’  R: 5’-GGTTTCACCTCCTGCTCTAAA-3’ | IDT-Primer Quest |
| HK1 | F: 5’-CACATTGATCTGGTGGAAGGA-3’  R: 5’-CTCTGTCCGGATGTCTTCTAATG-3’ | IDT-Primer Quest |
| HK2 | F: 5’-AGCCACCACTCACCCTACTGC-3’  R: 5’-CTGGAGCCCATTGTCCGTTAC-3’ | Chen *et al*., 2014 |
| PKM2 | F: 5′-ATTATTTGAGGAACTCCGCCGCCT-3′  R: 5′-ATTCCGGGTCACAGCAATGATGG-3′ | Goldberg and Sharp, 2012 |
| LDHA | F: 5’-ACCCAGATTTAGGGACTGATAAAG-3’  R: 5’-CCAATAGCCCAGGATGTGTAG-3’ | IDT-Primer Quest |
| HIF-1α | F: 5’-GAACGTCGAAAAGAAAAGTCTCG-3’  R: 5’-CCTTATCAAGATGCGAACTCACA-3’ | IDT-Primer Quest |
| c-MYC | F: 5’-GGCTCCTGGCAAAAGGTCA-3’  R: 5’-CTGCGTAGTTGTGCTGATGT-3’ | Primer Bank |
| p53 | F: 5’-CAGCACATGACGGAGGTTG-3’  R: 5’-TCATCCAAATACTCCACACGC-3’ | IDT-Primer Quest |

Chen J, Zhang S, Li Y, Tang Z, Kong W. Hexokinase 2 overexpression promotes the proliferation and survival of laryngeal squamous cell carcinoma. Tumour Biol. 2014;35(4):3743-53.

Goldberg MS, Sharp PA. Pyruvate kinase M2-specific siRNA induces apoptosis and tumor regression. J Exp Med 2012;209(2):217-24.
