# Supplementary material for: Clinicopathologic Features and Molecular Characteristics of Glucose Metabolism Contributing to ¹⁸F-fluorodeoxyglucose Uptake in Gastrointestinal Stromal Tumors
Source: PLoS One. 2015 Oct 28;10(10):e0141413. doi: 10.1371/journal.pone.0141413 (PMC4625049; doi:10.1371/journal.pone.0141413)
Supplement: S3 Table — (DOCX) [file pone.0141413.s007.docx]

**S3 Table.** Demographic and clinicopathologic features of 40 patients with GISTs.

|  |  |  | **Tumor** | | | | |  | **Immunohistochemistry** | |  | **Mutation status** |
| --- | --- | --- | --- | --- | --- | --- | --- | --- | --- | --- | --- | --- |
| Case No. | Age, years | Sex | Size (cm) | Mitotic count  (/50HPF) | Site | Risk | SUVmax |  | KIT | CD34 |  | *KIT* |
| 1 | 66 | Male | 7 | 15 | Stomach | High | 5.7 |  | + | - |  | K550_V555 del |
| 2 | 46 | Female | 5 | 12 | Jejunum | High | 4.5 |  | + | + |  | M552_D572 del |
| 3 | 71 | Male | 7.5 | 22 | Stomach | High | 5.2 |  | + | - |  | V559D |
| 4 | 67 | Male | 8.5 | 16 | Stomach | High | 12.4 |  | + | + |  | M552_Y553 del |
| 5 | 65 | Female | 9 | 20 | Ileum | High | 16.9 |  | + | - |  | Y503_F504 ins AY |
| 6 | 58 | Male | 11 | 12 | Stomach | High | 12.5 |  | + | + |  | K558_E562 del |
| 7 | 65 | Female | 13 | 13 | Jejunum | High | 19.2 |  | + | - |  | M552_I571 del |
| 8 | 53 | Male | 4.5 | 11 | Stomach | High | 8.9 |  | + | + |  | W557_K558 del |
| 9 | 56 | Male | 9 | 14 | Jejunum | High | 9.2 |  | + | - |  | *wild* |
| 10 | 77 | Male | 6.5 | 11 | Ileum | High | 14.7 |  | + | + |  | Y503_F504 ins AY |
| 11 | 54 | Male | 12 | 8 | Stomach | High | 12.0 |  | + | + |  | V559D |
| 12 | 71 | Male | 20 | 12 | Stomach | High | 21.4 |  | + | + |  | Q556_K558 del |
| 13 | 58 | Female | 6 | 60 | Rectum | High | 8 |  | + | + |  | *wild* |
| 14 | 49 | Male | 9 | 8 | Jejunum | High | 5.4 |  | + | + |  | Y568_L576 del |
| 15 | 66 | Male | 13 | 48 | Stomach | High | 14.5 |  | + | + |  | W557_K558 del |
| 16 | 27 | Male | 8 | 6 | Jejunum | High | 14.2 |  | + | + |  | W557_V560 del |
| 17 | 60 | Female | 7 | 12 | Stomach | High | 3.2 |  | + | + |  | D579 del |
| 18 | 35 | Female | 8.5 | 152 | Ileum | High | 6.7 |  | + | - |  | Q556_V560 del |
| 19 | 65 | Female | 17 | 70 | Stomach | High | 13 |  | + | + |  | *wild* |
| 20 | 69 | Female | 9 | 4 | Stomach | Intermediate | 8.1 |  | + | + |  | K558_I563 del |
| 21 | 22 | Female | 3.5 | 10 | Stomach | Intermediate | 5.0 |  | + | + |  | T574_K581 del |
| 22 | 73 | Female | 2.5 | 10 | Stomach | Intermediate | 4.9 |  | + | + |  | W557R |
| 23 | 83 | Female | 6.5 | 4 | Stomach | Intermediate | 2.4 |  | + | + |  | *wild* |
| 24 | 67 | Female | 5.8 | 5 | Duodenum | Intermediate | 9.5 |  | + | + |  | Y503_F504 ins AY |
| 25 | 75 | Male | 7.3 | 0 | Jejunum | Intermediate | 2.9 |  | + | + |  | *wild* |
| 26 | 49 | Female | 7 | 4 | Stomach | Intermediate | 2.9 |  | + | + |  | *wild* |
| 27 | 54 | Female | 5.5 | 2 | Duodenum | Intermediate | 7.9 |  | + | - |  | W557_K558 del |
| 28 | 64 | Female | 3.7 | 3 | Ileum | Low | 10.1 |  | + | + |  | Y503_F504 ins AY |
| 29 | 53 | Female | 2.8 | 2 | Jejunum | Low | 2 |  | + | - |  | *wild* |
| 30 | 20 | Female | 2.3 | 3 | Duodenum | Low | 3.9 |  | + | - |  | W557_K558 del |
| 31 | 58 | Male | 5 | 1 | Jejunum | Low | 2.2 |  | + | + |  | V560 del |
| 32 | 69 | Male | 5 | 4 | Jejunum | Low | 4.8 |  | + | - |  | *wild* |
| 33 | 52 | Female | 2.5 | 1 | Duodenum | Low | 4.3 |  | + | - |  | V560D |
| 34 | 75 | Male | 3 | 4 | Duodenum | Low | 2.9 |  | + | - |  | V559D |
| 35 | 59 | Male | 2.7 | 2 | Duodenum | Low | 1.8 |  | + | + |  | D572Y |
| 36 | 57 | Female | 2.3 | 1 | Stomach | Low | 2.6 |  | + | + |  | L576P |
| 37 | 62 | Female | 3.5 | 3 | Stomach | Low | 3.7 |  | + | + |  | L576P |
| 38 | 65 | Female | 3.8 | 0 | Duodenum | Low | 2.6 |  | + | + |  | W557_K558 del |
| 39 | 64 | Male | 3.5 | 1 | Stomach | Low | 2.4 |  | + | + |  | V560D |
| 40 | 49 | Female | 2 | 2 | Jejunum | Low | 12.2 |  | + | + |  | W557_K558 del |
